# Composition of Particulate Matter and Bacterial Community in Gut Contents and Surrounding Sediments of Three Sipunculan Species (*Siphonosoma australe*, *Phascolosoma arcuatum*, and *Sipunculus nudus*)

**DOI:** 10.3390/ijms24066001

**Published:** 2023-03-22

**Authors:** Chunsheng Liu, Chuang Liu, Fei Gao, Aimin Wang, Haiqing Wang, Yumei Yang, Linwen He

**Affiliations:** 1 State Key Laboratory of Marine Resource Utilization in South China Sea, Hainan University, Haikou 570228, China; 2 College of Marine Science, Hainan University, Haikou 570228, China

**Keywords:** sipunculan worms, grain size, total organic matter, 16S ribosomal RNA, bacterial community composition

## Abstract

*Siphonosoma australe*, *Phascolosoma arcuatum,* and *Sipunculus nudus* are three important sipunculan species in tropical intertidal zones. In this study, the particle size, organic matter content, and bacterial community composition in the gut contents of three different sipunculans and their surrounding sediments were analyzed. The grain size fractions of sipunculans’ guts were significantly different from those of their surrounding sediments; particle size fractions < 500 μm were favored by the sipunculans. As for the total organic matter (TOM), higher contents of organic matter were observed in the guts than in the surrounding sediments in all three sipunculan species. The bacterial community composition of all the samples was investigated by 16S rRNA gene sequencing, in which a total of 8974 OTUs were obtained from 24 samples based on a 97% threshold. The predominant phylum identified from the gut contents of three sipunculans was Planctomycetota, while the predominant phylum in their surrounding sediments was Proteobacteria. At the genus level, the most abundant genus was *Sulfurovum* (average 4.36%) in the surrounding sediments, while the most abundant genus was *Gplla* (average 12.76%) in the gut contents. The UPGMA tree showed that the samples from the guts of three different sipunculans and their surrounding sediments were clustered separately into two groups, which showed that these three sipunculans had a different bacterial community composition with their surrounding sediments. The grain size and total organic matter (TOM) had the greatest impacts on the bacterial community composition at both the phylum and genus levels. In conclusion, the differences in particle size fractions, organic matter content, and bacterial community composition between the gut contents and surrounding sediments in these three sipunculan species might be caused by their selective ingestion.

## 1. Introduction

Sipunculans, commonly named peanut worms, are a group of non-segmented coelomic animals living in different oceanic habitats, from intertidal to abyssal zones [1]. There are about 150 species in the Sipuncula all over the world, and 39 species are identified in China [1,2]. Due to their economic and nutritional values, some of them have become important culture species in China. *Siphonosoma australe* is a local fishery resource purchased in Hainan Island, and this species is often found in brackish waters (Pagola-Carte and Saiz-Salinas, 2000). *Phascolosoma arcuatum* is widely distributed in intertidal zones of East and South China, and it is a newly developed mariculture species [3,4]. *Sipunculus nudus* distributes widely along the southern coast of China and has been cultured successfully in China for decades [5].

Aside from the economic values, the sipunculans also play an important role in benthic ecosystems through bioturbations [6,7]. Most sipunculans are deposit feeders; they consume detritus and fecal material as well as bacteria, algae, and small invertebrates [8,9]. The characteristics of sediments change with the processes of benthic organisms through burrowing, feeding, and excretion, which might affect the bacterial communities in sediments [7,10]. For example, Aller [11] showed that the bacterial population and activity increased in sediments immediately surrounding burrows inhabited by bottom-dwelling animals compared to the ambient sediments; the bioturbation of *S. nudus* also significantly affected the bacterial community composition of intertidal sediments from the surface to the bottom layer [7].

Many studies have revealed that the gut bacterial population of invertebrates are different from their habitat environment [12,13,14]. The gut bacteria can aid the digestion of deposit-feeding holothurians, and the composition changes of bacteria are concerned with the organic matter content in sediments [15]. In tropical sea cucumbers, clear differences in the bacterial community composition between the gut content and ambient sediment were found [16]. In *P. aff. Turnerae,* the distinctive bacterial community structure was discovered when compared to those associated with the sediment [17].

*S. australe, P. arcuatum,* and *S. nudus* are three important edible sipunculans in intertidal zones of Hainan Island [1,18]. Until now, the research on the bacterial community of gut contents and surrounding sediments of *S. australe* and *P. arcuatum* had not been studied. Only a few reports exist on the gut bacterial community structure of *S. nudus.* For example, Ouyang et al. [19] identified bacterial groups in the gut of *S. nudus* through culture-independent methods, and they found that *Desulfovibrio, Desulfobacterium, Desulfomonile,* and *Prolixibacter* were predominant bacteria. Li et al. [7] analyzed the bacterial community composition of sediments from different layers in the *S. nudus* farming zone, and the bacterial community composition was changed by the bioturbation of *S. nudus*. Zhong et al. [20] reported that Proteobacteria were the dominant bacterial communities in the intestine, coelomic fluid, and the culture environment of *S. nudus*, while the other dominant phyla were highly variable. The microbial communities in the intestine of *S. nudus* and three different surrounding sediments were analyzed by Li et al., and they found that the activity of *S. nudus* affected the bacterial community in sediments [21]. Therefore, it is necessary to compare the bacterial community compositions in the digestive tract of these three sipunculan species and their surrounding sediments.

In this study, the particle size, organic matter content, and bacterial community composition in gut contents of three different sipunculans and their surrounding sediments were examined. Furthermore, the correlations between the bacterial community composition and grain size fraction/TOM were also detected. The results might be helpful in revealing the bacterial community diversity and abundance in different sipunculans and their surrounding sediments, and the reasons for the bacterial community differences.

## 2. Results

### 2.1. Grain Size Fraction and TOM of Gut Contents and Surrounding Sediments

The most abundant grain size fractions of SAE (surrounding sediments of *S. australe*) were 250–500 μm and 500–1000 μm, which accounted for 29.59 ± 0.36% and 28.92 ± 2.69%, respectively. The main grain size fractions of SAI (gut contents of *S. australe*) were 125–250 μm and 63–125 μm (accounting for 41.33 ± 2.15% and 27.33 ± 1.09%, respectively), which were significantly different to those of SAE (*p* < 0.05). There were no particle size fractions > 1000 μm in the gut of *S. australe* (Table 1).

For the *P. arcuatum,* the grain size fractions of 250–500 μm and 125–250 μm in sediments accounted for 35.53 ± 1.40% and 28.03 ± 2.14%, respectively, while the highest fraction size was 125–250 μm in its gut (42.36 ± 2.22%). The particle size fractions > 1000 μm were also not found in the gut of *P. arcuatum* (Table 1).

As for *S. nudus,* though the main grain sizes for both guts and sediments were 250–500 μm and 125–250 μm, respectively, significant differences in grain fractions in 250–500 μm and 125–250 μm were observed (*p* < 0.05). Furthermore, particle size fractions > 1000 μm were found in *S. nudus* guts (Table 1).

**Figure 1:**
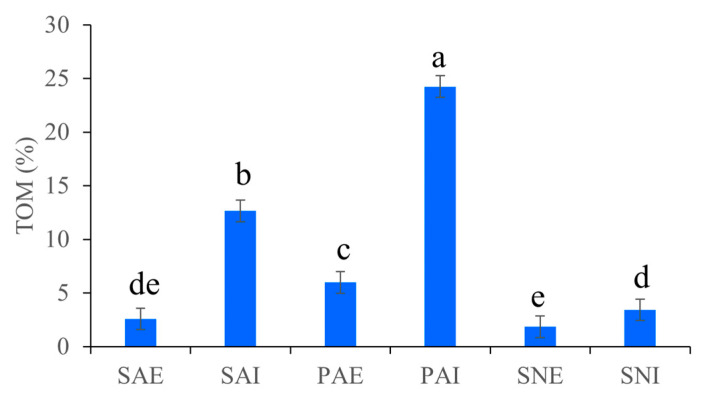
Total organic matter (TOM) content in gut contents and surrounding sediments of *S. australe, P. arcuatum,* and *S. nudus*. The different letters indicate significant differences among samples (*p* < 0.05). The gut contents and surrounding sediments of *S. australe, P. arcuatum,* and *S. nudus* are, respectively, marked as SAI, PAI, SNI, SAE, PAE, and SNE.

As for TOM, a significantly higher content of organic matter was observed in the gut as opposed to those in the surrounding sediments in all the three sipunculans (*p* < 0.05, Figure 1). Among the three different sediments, the PAE (surrounding sediments of *P. arcuatum*) had the highest TOM (5.98%), which was significantly higher than those of SAE and SNE (surrounding sediments of *S. nudus*) (*p* < 0.05). When comparing the TOM in sipunculans’ guts, the values of PAI (gut contents of *P. arcuatum*) were highest, followed by SAI and SNI (gut contents of *S. nudus*), and significant differences were observed among them (*p* < 0.05). The ratio of organic matter content in the gut to surrounding sediments was highest in *S. australe* (4.92), followed by *P. arcuatum* (4.05) and *S. nudus* (1.86).

### 2.2. Bacterial Community of Gut Contents and Surrounding Sediments

After quality control, a total of 1,623,045 high-quality sequences were obtained and used for further analysis. Each sample sequence’s number ranged from 30,280 to 95,420, with an average of 67,628. A total of 2876 OTUs were obtained from 24 samples based on a 97% threshold (Table S1). High-throughput raw sequence data were deposited in the US national center for biotechnology information (NCBI) GenBank short read archive (SRA) under the accession numbers SAMN33329954-SAMN33329977.

**Figure 2:**
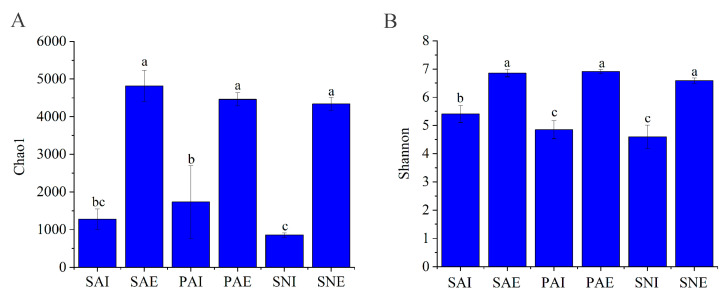
α-diversity of microbial communities in gut contents and surrounding sediments of *S. australe, P. arcuatum,* and *S. nudus*. (**A**) for Chao1 index, (**B**) for Shannon diversity index. The different letters indicate significant differences among samples (*p* < 0.05). The gut contents and surrounding sediments of *S. australe, P. arcuatum,* and *S. nudus* are, respectively, marked as SAI, PAI, SNI, SAE, PAE, and SNE.

The Chao1 index was calculated to estimate the bacterial community richness of different sipunculans and their surrounding sediments. The Chao1 values did not have significant differences among three different surrounding sediments, while the Chao1 values of surrounding sediments were significantly higher than those of sipunculans’ gut contents, which indicated that a higher bacterial richness existed in surrounding sediments. The Chao1 for PAI was significantly higher than that of SNI, demonstrating that the gut of *P. arcuatum* had a higher bacterial richness than that of *S. nudus* (Figure 2).

The Shannon diversity index in surrounding sediments was significantly higher than that in the gut content samples. No significant difference was found between PAI and SNI; the Shannon diversity index of SAI was higher than those of PAI and SNI (Figure 2). The results showed that the bacterial diversity in surrounding sediments was higher than that in the gut of sipunculans, and the bacterial diversity of *S. nudus* was highest among three different sipunculans.

**Figure 3:**
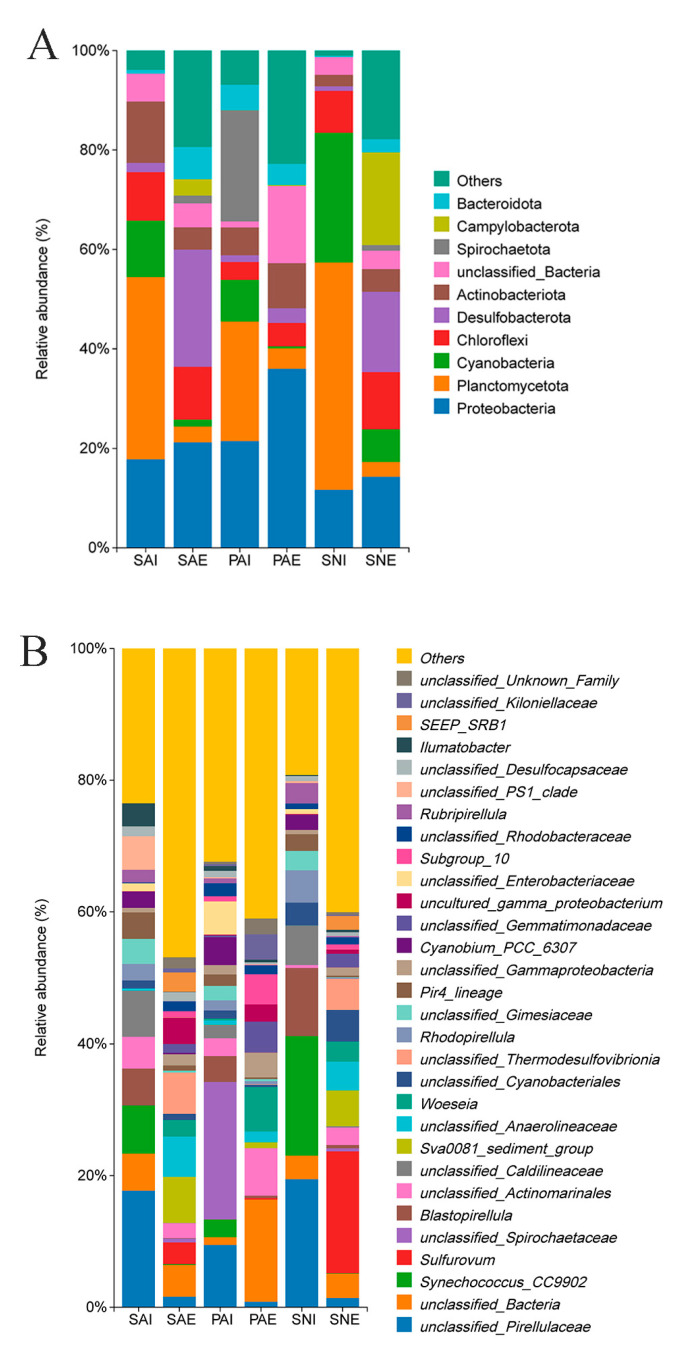
Relative abundances of bacterial communities at the phylum (**A**) and genus (**B**) level of the different groups. The gut contents and surrounding sediments of *S. australe, P. arcuatum,* and *S. nudus* are, respectively, marked as SAI, PAI, SNI, SAE, PAE, and SNE.

Fourteen bacteria phyla were detected in the 24 samples (average relative abundance > 0.1%); Proteobacteria and Planctomycetota were the most abundant phyla (39.21%); Cyanobacteria, Chloroflexi, Desulfobacterota, Actinobacteriota, Spirochaetota, Campylobacterota, Bacteroidota, Acidobacteriota, Firmicutes, Nitrospirota, Gemmatimonadota, and Myxococcota were also detected in the samples (Figure 3A and Table S2). In the surrounding sediments of *S. australe*, *P. arcuatum,* and *S. nudus*, the most abundant phylum was Proteobacteria (with relative abundances of 22.00%, 35.80%, and 14.37%, respectively, Table S3). Planctomycetota was the most abundance phylum in their gut contents (with relative abundances of 36.79%, 21.45%, and 45.49% for SAI, PAI, and SNI, respectively). Proteobacteria was the second most abundant phylum in the gut contents of three sipunculan species (average 16.34%). Cyanobacteria accounted for 11.52%, 8.56%, and 26.35% in SAI, PAI, and SNI, respectively (Table S3).

At the genus level, 13.65–41.55% of the reads in 24 samples were classified into 10 known genera (Figure 3B and Table S4). The ten most abundant genera (except the unclassified) in SAI, SAE, PAI, PAE, SNI, and SNE accounted for 24.13%, 14.97%, 15.13%, 13.64%, 41.55%, and 29.35%, respectively. The most abundant genus was *Sulfurovum* (average 7.45%) in the surrounding sediments, followed by *Sva0081*, *Woeseia,* and *Subgroup_10* (>1%). As for gut content, the most abundant genus was *Synechococcus* (average 9.49%), and the relative abundances of *Blastopirellula*, *Cyanobium*, *Rhodopirellula*, *Pir4,* and *Rubripirellula* were also more than 1% (Table S5).

**Figure 4:**
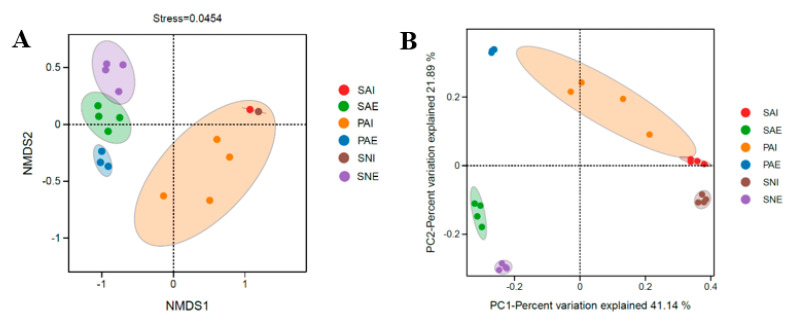
Nonmetric multidimensional scaling (NMDS) plot (**A**) and principal coordinates analysis (PCoA) plot (**B**) based on unweighted-unifrac distance showing the relatedness of the bacterial community composition between different samples. The gut contents and surrounding sediments of *S. australe, P. arcuatum,* and *S. nudus,* are, respectively, marked as SAI, PAI, SNI, SAE, PAE, and SNE.

**Figure 5:**
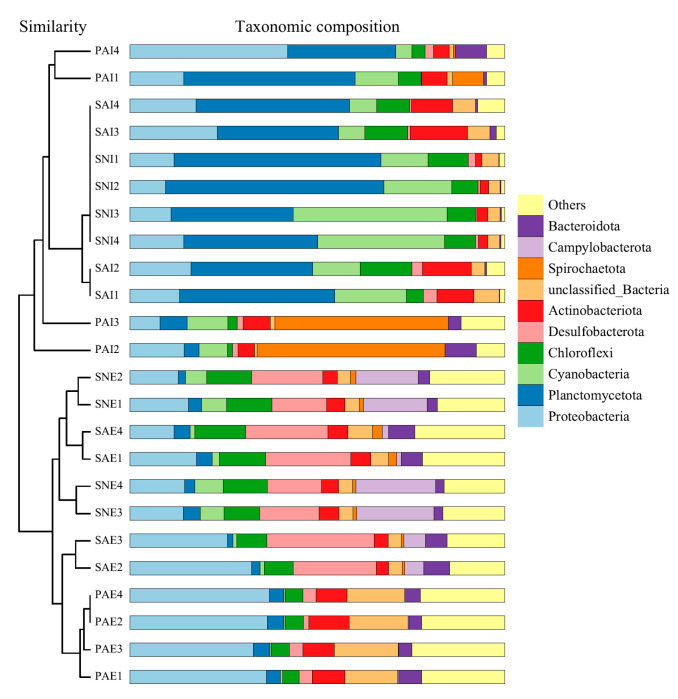
UPGMA tree showing the similarity of bacterial community structures among the different samples. The gut contents and surrounding sediments of *S. australe, P. arcuatum,* and *S. nudus* are, respectively, marked as SAI, PAI, SNI, SAE, PAE, and SNE.

NMDS and PCoA analyses were performed to explain the relationships between the bacterial community of different samples. NMDS analysis, based on the unweighted UniFrac distances, showed that the bacterial communities were clearly separated into four groups, which included SAE, PAE, SNE, and the gut contents of sipunculans (Figure 4A). The bacterial community composition of sipunculans’ gut contents overlapped with each other, indicating they had similar bacterial communities. In both NMDS and PCoA plots, the gut content samples of three different sipunculans were clustered separately from their own surrounding sediment samples (Figure 4). The UPGMA tree showed that the samples from the guts of three different sipunculans and their surrounding sediments were clustered separately into two groups (Figure 5), which showed that the sipunculans had different bacterial community compositions with the surrounding sediments.

**Figure 6:**
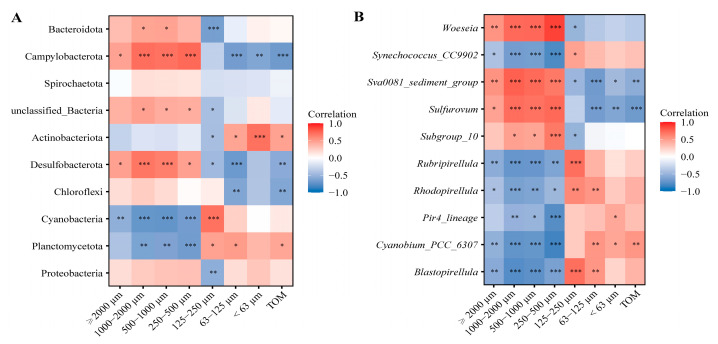
Heatmap of the correlations between the bacterial community composition (phylum (**A**) and genus (**B**)) and grain size fraction and TOM. Pearson correlation coefficient is displayed by the color of each cell in heatmap. A significant correlation is confirmed if the *p*-value with Bonferroni adjusted is less than 0.05 (*), 0.01 (**), or 0.001 (***).

The relationship between the bacterial community composition and grain size fraction/TOM was calculated in this study and the results are shown in Figure 6. Both grain size and TOM had the greatest impacts on the bacterial community composition at phylum and genus levels. At the phylum level, the contents of small grain sizes (<125 μm) and TOM showed a significantly positive relationship with Actinobacteriota and Planctomycetota (*p* < 0.05), and a negative relationship with Campylobacterota (*p* < 0.01); the contents of large grain sizes (≥250 μm) showed a significantly positive relationship with Campylobacterota and Desulfobacterota (*p* < 0.05), and a negative relationship with Cyanobacteria and Planctomycetota (*p* < 0.01). At genus level, the contents of small grain sizes (<125 μm) and TOM were found to positively correlate with *Cyanobium_PCC_6307* (*p* < 0.05), and to negatively correlate with *Sulfurovum* and *Sva0081_sediment_group* (*p* < 0.05). Furthermore, the contents of large grain sizes (≥250 μm) showed positive relationships with *Woeseia*, *Sva0081_sediment_group*, *Sulfurovum* (except at ≥2000 μm), and *Subgroup_10* (*p* < 0.05), while showing a negative relationship with the others, except *Pir4_lineage* at ≥2000 μm (*p* < 0.05).

## 3. Discussion

### 3.1. Habitat Differences of Three Sipunculan Species

Sipunculans inhabit a variety of habitats, including mud, sand, rocks, rhizomes of seagrasses, and living and dead shells, from intertidal to abyssal zones [6,18,22]. In this study, the sediment size distribution and composition of three sipunculan species were varied. Furthermore, the TOM contents in PAE were significantly higher than those in SAE and SNE. Therefore, we could conclude that the living habitats of *S. australe*, *P. arcuatum*, and *S. nudus* were different. Similarly, in a previous study, the different habitat preferences of these three sipunculan species were reported. For example, *S. australe* inhabits sandy and muddy bottoms of the intertidal and shallow subtidal zones [18]; *P. arcuatum* is a semiterrestrial sipunculan able to live in the mud of the mangrove swamp and can tolerate wide salinity and temperature changes [8,23]; *S. nudus* usually inhabits the intertidal zone and prefers sandy bottoms [2].

The PCoA analysis and UPGMA tree showed that the bacterial communities of three sipunculans’ surrounding sediments were clearly separated into three groups, which meant significant differences between the bacterial communities in these three sediments. As a mixing zone between marine and terrestrial habitats, the bacterial composition and abundance of intertidal sediment are various, and their changes often occur by many environmental factors, such as temperature, salinity, total organic carbon, SO_4_^2−^, and total phosphorus [24,25,26]. The differences in bacterial communities in the surrounding sediments further confirmed the conclusion that the living habitats of these three sipunculan species were different.

### 3.2. Comparison of Gut Microbe Composition among Three Sipunculan Species

Intertidal zones have a periodically changing environment due to the daily tide, which affect the physical and chemical conditions such as temperature, salinity, and light intensity [27]. The bacterial community in the intertidal sediments is significantly different to that of marine sediments [28,29]. Wang et al. [24] compared the levels of bacterial diversity in intertidal sediment and marine sediment, and a higher taxon richness and evenness were found in intertidal sediment. The higher Chao1 and Shannon diversity index were detected in the surrounding sediments compared to the gut content of three sipunculan species. Compared to the intestines of aquatic animals, the bacterial communities of sediments usually have higher diversity [16,21].

While comparing the bacterial composition in gut contents, the most abundant phylum in these detected sipunculan species was Planctomycetota. Planctomycetota can utilize polysaccharides as carbon and energy sources, and a significant proportion of Planctomycetota was found in the gut microbiome of fish and shrimp [30,31]. A large proportion of Cyanobacteria was found in SNI compared to SAI and PAI (*p* < 0.05, Table S3). Cyanobacteria might be an important food for sipunculans. The biomass of Cyanobacteria in the aquaculture tidal flat was found to reduce by the feeding of *S. nudus* [7]. At the genus level, *Synechococcus* (a group of Cyanobacteria, also named *GpIIa*) was the most abundant in the gut of sipunculans, and the abundance of *Synechococcus* in SNI was significantly higher than those of SAI and PAI (Table S5). Li et al. also found that the *S. nudus* intestine was enriched in *Synechococcus*, which might be an important food source for *S. nudus* [21]. The abundance of Actinobacteriota in the three species was significantly different (*p* < 0.05, Table S3). Actinomycetes play an important role in the degradation of organic matter [32], which might be important in the digestion of sipunculans [21].

Furthermore, the bacterial composition of *S. australe* was much closer to those of *S. nudus* than *P. arcuatum*. The habitat conditions may affect the bacterial community of sipunculans. In this study, *S. australe* and *S. nudus* were collected from sandy bottoms in the intertidal zone, while *P. arcuatum* was collected from muddy bottoms. Though these three sipunculan species had different habitat preferences, more similar habitats between *S. australe* and *S. nudus* were observed according to the TOM contents and bacterial community (analyzed by NMDS and PCoA) in their surrounding sediments. Therefore, similar bacterial communities were found in the gut of *S. australe* and *S. nudus,* though they were collected from a different place on Hainan Island.

### 3.3. Selective Feeding of Three Sipunculan Species

It has been reported that the deposit feeder ingested selectively when they fed [33,34,35]. In the present study, the main grain size in *S. australe* and *P. arcuatum* guts significantly decreased, compared to the surrounding sediments; the TOM contents in *S. australe* and *P. arcuatum* guts also significantly increased and the ratios of their TOM contents of guts to surrounding sediments were 4.92 and 4.05, respectively. These results suggested that *S. australe* and *P. arcuatum* had an obvious feeding selectivity to surrounding sediments. Hansen [36] reported that the food uptake of sipunculans was selective with regard to small grain sizes, which showed the same results as our study in *S. australe* and *P. arcuatum*. In a previous study, *S. nudus* was considered as having no selectivity to surrounding particles, because similar granulometric compositions were observed between the sand and intestinal contents of *S. nudus* [37]. However, in our study, though the main grain sizes in both guts and sediments of *S. nudus* were the same, the ratios of small-size particulate matter (grain size at 125–250 μm and 63–125 μm) and TOM content in guts were significantly increased compared to surrounding sediments. Therefore, we inferred that *S. nudus* had a weaker selective feeding compared to the other two sipunculan species.

Three different possible interactions existed between deposited feeders and sediment microbes: deposit-feeders compete for food with microorganisms, they feed the microbial community directly, or they synergistically interact [38]. *S. australe*, *P. arcuatum,* and *S. nudus* all belong to deposited feeders. It has been reported that *S. nudus* could transport organic matter from the surface layer into the bottom sediments, and its burrowing, feeding, and excretion processes reshape the bacterial community of sediments [2,7]. The activity of *S. nudus* has a significant influence on the bacterial community. In *P. aff. Turnerae* (a deep sea sipunculan) reported by Rubin-Blum et al. [17], a distinct proteobacterial microbiota was found in the gut, which could provide fixed carbon and detoxify sulfide, and help *P. aff. Turnerae* thrive in the deep-sea environment. In this study, the predominant phylum in the gut contents was Planctomycetota, while the predominant phylum in surrounding sediments was Proteobacteria. Proteobacteria was the most dominant phylum in sediments [24,39,40]. Planctomycetota are ubiquitous in many different environments, and they have been found in many eukaryotic organisms, such as sponges, ascidians, corals, and prawns [41,42]. The distinctive bacterial composition in the gut contents and surrounding sediments might have been caused by the selective ingestion of sipunculans.

## 4. Materials and Methods

### 4.1. Sample Collection

*Siphonosoma australe* and *Phascolosoma arcuatum* were collected from a natural distribution area in the intertidal zone of Changhua River in Changjiang, Hainan Province, China (*n* = 4). The sediments surrounding the animals were also collected for analysis (*n* = 4). The water temperature and salinity at the sampling site were 28.0 °C and 26‰, respectively.

*Sipunculus nudus* was collected from the intertidal zone of Guangcun in Danzhou, Hainan Province, China (*n* = 4). The sediments surrounding the animals were also collected for analysis (*n* = 4). The water temperature and salinity at the sampling site were 28.0 °C and 33‰, respectively.

All samples were stored on ice and transported to the laboratory for no more than 6 h. Each sipunculan individual was dissected under sterile conditions. In detail, worms were washed with sterile seawater, and the scarfskin was sterilized with 75% ethanol to reduce exogenous bacterial contamination. The ventral body walls were incised using a sterile scalpel to expose the body cavity, then all the gut contents were collected in sterile 2 mL tubes and stored at −80 °C for analysis. The gut contents and surrounding sediments of *S. nudus, P. arcuatum,* and *S. australe* were, respectively, marked as SNI1-4, PAI1-4, SAI1-4, SNE1-4, PAE1-4, and SAE1-4.

The particle sizes of gut contents and surrounding sediments were measured using a fine-sieve filtration method. The total organic matter (TOM) content was determined gravimetrically in a muffle furnace (Lichen Sx2–2.5-10, Shanghai, China) by incineration at 550 °C for 24 h.

### 4.2. DNA Extraction and PCR Amplification

The DNA of gut contents and surrounding sediments were extracted using the Soil DNA Kit (Omega Bio-Tek, Norcross, GA, USA) following the manufacturer’s instructions. The bacterial 16S rRNA genes’ V3–V4 region was amplified using the universal primer pairs 341F (CCTACGGGNGGCWGCAG) and 805R (GACTACHVGGGTATCTAATCC). The PCR conditions were: 98 °C for 1 min, 98 °C for 10 s, 50 °C for 30 s, 72 °C for 30 s (30 cycles), and lastly 72 °C for 5 min. The PCR products were mixed in equal-density ratios and then purified with the Qiagen gel extraction kit (Qiagen, Hilden, Germany).

### 4.3. Library Preparation and Sequencing

Sequencing libraries were generated using the TruSeq^®^ DNA PCR-Free sample preparation kit following the instructions. The library quality was evaluated on the Qubit@ 2.0 fluorometer (Thermo Fisher Scientific, Waltham, MA, USA) and Agilent Bioanalyzer 2100 device. Paired-end 2 × 250 bp sequencing was performed using the Illumina MiSeq platform at Sangon Biotechnology (Shanghai, China) Co. Ltd. (Shanghai, China).

### 4.4. Bioinformatic and Statistical Analysis

Paired-end reads were assigned to different samples based on their specific barcodes, and then the barcode and primer sequence were cut. According to the overlap relationship between paired-end reads, paired-end reads were merged using the software PEAR V0.9.8 [43]. The raw sequence reads were filtrated using PRINSEQ V0.20.4 [44]. After removing chimeric sequences by UCHIME V 4.2.40, sequence analysis was performed by Uparse V7.0.1001. Operational taxonomic units (OTUs) were selected (≥97%) and clustered using the default parameters, and the OTU counts less than 2 in all samples were filtered. The representative OTU sequences (most frequent sequences in each OTU) were selected and further analyzed with the Mothur algorithm and Silva database (release 138, http://www.arb-silva.de, accessed on 22 November 2022). Alpha-diversity indexes (Chao1, Shannon) were calculated by Quantitative Insights Into Microbial Ecology (QIIME V1.7.0) software [45]. The PCoA and NMDS analyses, based on the Unweighted UniFrac distance, were performed to analyze differences among multi-groups. Spearman correlation coefficients with the Bonferroni adjustment method were used to identify the correlations between the bacterial community composition (top ten most abundant at both phylum and genus levels) and grain size fraction/TOM.

## 5. Conclusions

In the present study, the ratio of the smaller grain size in *S. australe*, *P. arcuatum,* and *S. nudus* guts significantly increased, compared to the surrounding sediments. Accordingly, the TOM contents of guts in these three sipunculans showed a significant increase compared to their surrounding sediments. Furthermore, a lower bacterial richness and diversity, and different bacterial communities in these three sipunculans were observed compared to their surrounding sediments. In conclusion, the differences in particle size fractions, TOM content, and bacterial community composition between gut contents and surrounding sediments in these three sipunculans might be caused by their feeding selectivity.

## Figures and Tables

**Table 1:** Grain size fractions (%) in gut contents and surrounding sediments of *S. australe*, *P. arcuatum,* and *S. nudus*.

Grain Size	≥2000 μm	1000–2000 μm	500–1000 μm	250–500 μm	125–250 μm	63–125 μm	<63 μm
SAE	6.50 ± 1.40 ^a^	11.57 ± 2.45 ^a^	28.92 ± 2.69 ^a^	29.59 ± 0.36 ^b^	16.41 ± 1.10 ^e^	5.93 ± 1.26 ^c^	1.09 ± 0.49 ^d^
SAI	0.00 ± 0.00 ^d^	0.00 ± 0.00 ^d^	1.24 ± 0.33 ^e^	17.11 ± 1.23 ^d^	41.33 ± 2.15 ^c^	27.33 ± 1.09 ^a^	12.97 ± 0.34 ^a^
PAE	0.97 ± 0.36 ^b^	2.85 ± 1.02 ^b^	14.72 ± 2.63 ^b^	35.53 ± 1.40 ^a^	28.03 ± 2.14 ^d^	14.65 ± 1.51 ^b^	3.27 ± 1.01 ^c^
PAI	0.00 ± 0.00 ^d^	0.00 ± 0.00 ^d^	1.38 ± 0.43 ^e^	20.31 ± 1.12 ^c^	42.36 ± 2.22 ^c^	29.32 ± 2.10 ^a^	6.63 ± 0.68 ^b^
SNE	0.30 ± 0.16 ^c^	1.11 ± 0.09 ^b^	8.77 ± 1.01 ^c^	32.36 ± 1.74 ^ab^	51.11 ± 2.21 ^b^	6.25 ± 1.06 ^c^	0.10 ± 0.09 ^e^
SNI	0.50 ± 0.20 ^bc^	0.32 ± 0.11 ^c^	3.78 ± 0.077 ^d^	22.44 ± 1.43 ^c^	60.13 ± 1.17 ^a^	11.96 ± 1.34 ^b^	0.52 ± 0.21 ^de^

Note: The values are presented as the mean ± SD (*n* = 3). Values in columns with different letters indicate significant differences (*p* < 0.05). The gut contents and surrounding sediments of *S. australe, P. arcuatum,* and *S. nudus* are, respectively, marked as SAI, PAI, SNI, SAE, PAE, and SNE.

## Supplementary Materials

The following supporting information can be downloaded at: https://www.mdpi.com/article/10.3390/ijms24066001/s1.

## References

[1] Li F., Zhou H., Wang W. (1992). A checklist of Sipuncula from the China coasts. J. Ocean. Univ. Qingdao.

[2] Li J., Xie X., Zhu C., Guo Y., Chen S. (2017). Edible peanut worm (*Sipunculus nudus*) in the Beibu Gulf: Resource, aquaculture, ecological impact and counterplan. J. Ocean. Univ. China.

[3] Ying X., Dahms H., Liu X., Wu H., Zhang Y., Chen C., Zhou Z., Zeng G., Zhou K., Yang W. (2009). Development of germ cells and reproductive biology in the sipunculid *Phascolosoma esculenta*. Aquac. Res..

[4] Tan Q., Ke C., Wang W. (2013). Rapid assessments of metal bioavailability in marine sediments using coelomic fluid of sipunculan worms. Environ. Sci. Technol..

[5] Wang Y., Shi T., Huang G., Gong J. (2018). Molecular detection of eukaryotic diets and gut mycobiomes in two marine sediment-dwelling worms, *Sipunculus nudus* and *Urechis unicinctus*. Microbes Environ..

[6] Shields M.A., Kedra M. (2009). A deep burrowing sipunculan of ecological and geochemical importance. Deep Sea Res. Part I.

[7] Li J., Hu R., Guo Y., Chen S., Xie X., Qin J.G., Ma Z., Zhu C., Pei S. (2019). Bioturbation of peanut worms *Sipunculus nudus* on the composition of prokaryotic communities in a tidal flat as revealed by 16S rRNA gene sequences. MicrobiologyOpen.

[8] Cutler E.B. (1994). The Sipuncula: Their Systematics, Biology, and Evolution.

[9] Maiorova A., Adrianov A. Distribution of peanut worms (Sipuncula) in the west Pacific. Proceedings of the China-Russian bilateral Symposium on Comparison on Marine Biodiversity in the Northwest Pacific Ocean.

[10] Sokratis P., Trine G., Raymond P.C., Maria T., Erik K. (2005). Sediment properties and bacterial community in burrows of the ghost shrimp *Pestarella tyrrhena* (Decapoda: Thalassinidea). Aquat. Microb. Ecol..

[11] Aller R.C., Blackburn T.H., Soerensen J. (1988). Benthic fauna and biogeochemical processes in marine sediments: The role of burrow structures. Nitrogen Cycling in Coastal Marine Environments.

[12] Harris J.M. (1993). The presence, nature, and role of gut microflora in aquatic invertebrates: A synthesis. Microb. Ecol..

[13] Bogatyrenko E.A., Buzoleva L.S. (2016). Characterization of the gut bacterial community of the Japanese sea cucumber *Apostichopus japonicus*. Microbiology.

[14] Li F., Gao F., Tan J., Fan C., Sun H., Yan J., Chen S., Wang X. (2016). Characterization and identification of enzyme-producing microflora isolated from the gut of sea cucumber *Apostichopus japonicus*. Chin. J. Oceanol. Limn..

[15] Amaro T., Harry W., Herndl G.J., Cunha M.R., Billett D.S.M. (2009). Deep-sea bacterial communities in sediments and guts of deposit-feeding holothurians in Portuguese canyons (NE Atlantic). Deep Sea Res. Part I.

[16] Gao F., Zhang Y., Wu P., Chen M., He L., Xu Q., Wang A. (2022). Bacterial community composition in gut content and ambient sediment of two tropical wild sea cucumbers (*Holothuria atra* and *H. leucospilota*). J. Oceanol. Limnol..

[17] Rubin-Blum M., Shemesh E., Goodman-Tchernov B., Coleman D.F., Ben-Avraham Z., Tchernov D. (2014). Cold seep biogenic carbonate crust in the Levantine basin is inhabited by burrowing *Phascolosoma aff. turnerae*, a sipunculan worm hosting a distinctive microbiota. Deep Sea Res. Part I.

[18] Pagola-Carte S., Saiz-Salinas J.I. (2000). Sipuncula from Hainan Island (China). J. Nat. Hist..

[19] Ouyang Y.C., Chen F.L., Sun C.B., Zhou J.Z. (2012). Study on the diversity of bacteria from peanut worm (*Sipunculus nudus*). Guangdong Agric. Sci..

[20] Zhong R., Huang J., Liao Y., Yang C., Wang Q., Deng Y. (2022). Insights into the bacterial community compositions of peanut worm (*Sipunculus nudus*) and their association with the surrounding environment. Front. Mar. Sci..

[21] Li J., Chen S., Wu P., Zhu C., Hu R., Li T., Guo Y. (2023). Insights into the relationship between intestinal microbiota of the aquaculture worm *Sipunculus nudus* and surrounding sediments. Fishes.

[22] Açik S. (2017). Distribution of sipunculans along the Aegean and Levantine coasts of Turkey. Cah. Biol. Mar..

[23] Ip Y.K., Tan G.Q., Kuah S.S.L., Chew S.F. (1997). Detoxification of environmental sulfide to sulfane sulfur in the intertidal sipunculid *Phascolosoma arcuatum*. J. Comp. Physiol. B.

[24] Wang Y., Sheng H., He Y., Wu J., Jiang Y., Tam N.F., Zhou H. (2012). Comparison of the levels of bacterial diversity in freshwater, intertidal wetland, and marine sediments by using millions of Illumina tags. Appl. Environ. Microbiol..

[25] Zheng B., Wang L., Liu L. (2014). Bacterial community structure and its regulating factors in the intertidal sediment along the Liaodong Bay of Bohai Sea, China. Microbiol. Res..

[26] Guo X., Lu D., Niu Z., Feng J., Chen Y., Tou F., Liu M., Yang Y. (2018). Bacterial community structure in response to environmental impacts in the intertidal sediments along the Yangtze Estuary, China. Mar. Pollut. Bull..

[27] Menge B.A., Branch G.M., Bertness M.D., Gaines S.D., Hay M.E. (2001). Rocky intertidal communities. Marine Community Ecology.

[28] Lasher C., Dyszynski G., Everett K., Edmonds J., Ye W., Sheldon W., Wang S., Joye S.B., Moran M.A., Whitman W.B. (2009). The diverse bacterial community in intertidal, anaerobic sediments at Sapelo Island, Georgia. Environ. Microbiol..

[29] Lee H., Heo Y., Kwon S.L., Yoo Y., Kim D., Lee J., Kwon B.O., Khim J.S., Kim J.J. (2021). Environmental drivers affecting the bacterial community of intertidal sediments in the Yellow Sea. Sci. Total Environ..

[30] Kallscheuer N., Wiegand S., Kohn T., Boedeker C., Jeske O., Rast P., Müller R.-W., Brümmer F., Heuer A., Jetten M.S.M. (2020). Cultivation-independent analysis of the bacterial community associated with the calcareous sponge *Clathrina clathrus* and isolation of *Poriferisphaera corsica* Gen. Nov., Sp. Nov., belonging to the barely studied class Phycisphaerae in the phylum Planctomycetota. Front. Microbiol..

[31] Kohn T., Wiegand S., Boedeker C., Rast P., Heuer A., Jetten M., Schüler M., Becker S., Rohde C., Müller R.-W. (2020). *Planctopirus ephydatiae*, a novel Planctomycete isolated from a freshwater sponge. Sys. Appl. Microbiol..

[32] Sheikh M., Rathore D., Gohel S., Singh S. (2018). Marine Actinobacteriota associated with the invertebrate hosts: A rich source of bioactive compounds: A review. J. Cell Tissue Res..

[33] Taghon G.L., Jumars P.A. (1984). Variable ingestion rate and its role in optimal foraging behavior of marine deposit feeders. Ecology.

[34] Zamora L.N., Jeffs A.G. (2011). Feeding, selection, digestion and absorption of the organic matter from mussel waste by juveniles of the deposit-feeding sea cucumber, *Australostichopus mollis*. Aquaculture.

[35] Jumars P.A., Dorgan K.M., Lindsay S.M. (2015). Diet of worms emended: An update of polychaete feeding guilds. Ann. Rev. Mar. Sci..

[36] Hansen M.D. (1978). Nahrung und Freßverhalten bei Sedimentfressern dargestellt am Beispiel von Sipunculiden und Holothurien. Helgoländer Wiss. Meeresunters..

[37] Edmonds S.J. (1962). Some notes on the abundance, environment, and nutrition of *Sipunculus nudus* L. (Sipunculoidea) at Morgat, Brittany. Cah. Biol. Mar..

[38] Amon R.M.W., Herndl G.J. (1991). Deposit feeding and sediment I. interrelationship between *Holofhuria fubulosa* (Holofhurioida, Echinodermata) and the sediment microbial community. Mar. Ecol..

[39] Andrei A., Baricz A., Robeson M.S., Păuşan M.R., Tămaş T., Chiriac C., Szekeres E., Barbu-Tudoran L., Levei E.A., Coman C. (2017). Hypersaline sapropels act as hotspots for microbial dark matter. Sci. Rep..

[40] Shen H., Jiang G., Wan X., Li H., Qiao Y., Thrush S., He P. (2017). Response of the microbial community to bioturbation by benthic macrofauna on intertidal flats. J. Exp. Mar. Biol. Ecol..

[41] Lage O.M., Bondoso J. (2014). Planctomycetota and macroalgae, a striking association. Front. Microbiol..

[42] Fuerst J.A., Sagulenko E. (2011). Beyond the bacterium: Planctomycetota challenge our concepts of microbial structure and function. Nat. Rev. Microbiol..

[43] Zhang J., Kobert K., Flouri T., Stamatakis A. (2014). PEAR: A fast and accurate Illumina Paired-End reAd mergeR. Bioinformatics.

[44] Schmieder R., Edwards R. (2011). Quality control and preprocessing of metagenomic datasets. Bioinformatics.

[45] Caporaso J.G., Kuczynski J., Stombaugh J., Bittinger K., Bushman F.D., Costello E.K., Fierer N., Peña A.G., Goodrich J.K., Gordon J.I. (2010). QIIME allows analysis of high-throughput community sequencing data. Nat. Methods.

