# Supplementary material for: Composition of Particulate Matter and Bacterial Community in Gut Contents and Surrounding Sediments of Three Sipunculan Species (Siphonosoma australe, Phascolosoma arcuatum, and Sipunculus nudus)
Source: Int J Mol Sci. 2023 Mar 22;24(6):6001. doi: 10.3390/ijms24066001 (PMC10054262; doi:10.3390/ijms24066001)
Supplement: Supplementary file 1 [file ijms-24-06001-s001.zip › Table S3 Average relative abundances of top 10 phylum from different groups.pdf]

	Proteobacteria	Planctomycetota	Cyanobacteria	Chloroflexi	Desulfobacterota	Actinobacteriota	Unclassified _Bacteria	Spirochaetota	Campylobacterota	Bacteroidota
SAI	17.63±2.11^{b}	36.79±2.54^{a}	11.52±2.86^{b}	9.67±1.98^{a}	1.90±0.78^{c}	12.24±1.2^{a}	5.62±0.70^{b}	0.00±0.00^{b}	0.00±0.00^{c}	0.70±0.35^{c}
SAE	22.00±4.56^{b}	3.07±0.70^{c}	1.36±0.21^{c}	10.38±1.48^{a}	23.87±1.58^{a}	4.44±0.53^{c}	4.61±0.70^{bc}	1.49±0.49^{b}	3.44±1.13^{b}	6.36±0.37^{a}
PAI	19.75±7.61^{b}	21.45±9.79^{b}	8.56±1.64^{bc}	3.38±1.00^{b}	1.36±0.44^{c}	5.64±0.83^{c}	1.15±0.16^{d}	26.25±12.78^{a}	0.02±0.01^{c}	5.21±1.89^{ab}
PAE	35.80±0.98^{a}	4.13±0.19^{c}	0.41±0.04^{c}	4.73±0.08^{b}	3.01±0.52^{c}	9.01±0.60^{b}	15.58±0.64^{a}	0.01±0.01^{b}	0.20±0.04^{c}	4.23±0.65^{ab}
SNI	11.66±1.02^{b}	45.49±6.58^{a}	26.35±6.65^{a}	8.40±0.82^{a}	0.92±0.36^{c}	2.32±0.27^{d}	3.55±0.36^{c}	0.00±0.00^{b}	0.00±0.00^{c}	0.26±0.06^{c}
SNE	14.37±0.56^{b}	3.14±0.56^{c}	6.58±0.39^{bc}	11.31±0.61^{a}	16.02±1.03^{b}	4.66±0.30^{c}	3.70±0.07^{c}	1.13±0.10^{b}	18.85±1.19^{a}	2.60±0.20^{bc}

Values in column with different letters indicate significant differences ($p < 0.05$).
