# Supplementary material for: Composition of Particulate Matter and Bacterial Community in Gut Contents and Surrounding Sediments of Three Sipunculan Species (Siphonosoma australe, Phascolosoma arcuatum, and Sipunculus nudus)
Source: Int J Mol Sci. 2023 Mar 22;24(6):6001. doi: 10.3390/ijms24066001 (PMC10054262; doi:10.3390/ijms24066001)
Supplement: Supplementary file 1 [file ijms-24-06001-s001.zip › Table S5 Average relative abundances of top 10 genus from different groups.pdf]

	Blastopirellula	*Cyanobium_PCC_6307*	*Pir4_lineage*	*Rhodopirellula*	*Rubripirellula*	*Subgroup_10*	*Sulfurovum*	*Sva0081_sediment_group*	*Synechococcus_CC9902*	*Woeseia*
SAI	5.65±1.16^{b}	2.54±1.08^{ab}	4.01±0.52^{a}	2.52±0.31^{b}	1.94±0.25^{b}	0.03±0.02^{c}	0.00±0.00^{c}	0.00±0.00^{c}	7.44±2.04^{b}	0.00±0.00^{c}
SAE	0.08±0.04^{c}	0.22±0.07^{b}	0.73±0.25^{c}	0.02±0.02^{d}	0.05±0.03^{c}	0.85±0.22^{b}	3.43±1.13^{b}	6.85±0.88^{a}	0.15±0.05^{b}	2.59±1.03^{b}
PAI	3.50±1.59^{bc}	4.26±1.25^{a}	1.49±0.80^{bc}	1.36±0.59^{c}	0.67±0.25^{c}	0.85±0.27^{b}	0.02±0.01^{c}	0.02±0.01^{c}	2.64±0.38^{b}	0.33±0.12^{c}
PAE	0.38±0.06^{c}	0.04±0.01^{b}	0.24±0.01^{c}	0.48±0.05^{cd}	0.11±0.05^{c}	4.57±0.16^{a}	0.20±0.04^{c}	0.90±0.04^{c}	0.03±0.02^{b}	6.70±0.53^{a}
SNI	10.27±1.79^{a}	2.37±1.23^{ab}	2.54±0.34^{b}	4.90±0.60^{a}	3.08±0.69^{a}	0.00±0.00^{c}	0.00±0.00^{c}	0.00±0.00^{c}	18.4±6.16^{a}	0.00±0.00^{c}
SNE	0.56±0.19^{c}	0.05±0.02^{b}	0.02±0.05^{c}	0.20±0.06^{d}	0.21±0.05^{c}	0.77±0.03^{b}	18.72±1.15^{a}	5.37±0.46^{b}	0.07±0.01^{b}	3.15±0.39^{b}

Values in column with different letters indicate significant differences ($p < 0.05$).
